# Frequency, Severity and Impact of Pegylated Interferon Alpha–Associated Flares in Hepatitis D Infection

**DOI:** 10.1111/jvh.70022

**Published:** 2025-03-15

**Authors:** Svenja Hardtke, Cihan Yurdaydin, Florin A. Caruntu, Manuela G. Curescu, Kendal Yalcin, Ulus S. Akarca, Selim Gürel, Stefan Zeuzem, Andreas Erhardt, Stefan Lüth, George V. Papatheodoridis, Kerstin Port, Michael P. Manns, Markus Cornberg, Julia Kahlhöfer, Heiner Wedemeyer

**Affiliations:** ^1^ Department of Gastroenterology, Hepatology, Infectious Diseases, and Endocrinology Hannover Medical School Hannover Germany; ^2^ German Center for Infectious Disease Research (DZIF) HepNet Study‐House/German Liver Foundation Hannover Germany; ^3^ University Hospital Hamburg Eppendorf Institute for Infection Research and Vaccine Development Hamburg Germany; ^4^ Department of Gastroenterology Ankara University Medical School Ankara Turkey; ^5^ Department of Gastroenterology & Hepatology Koc University Medical School Istanbul Turkey; ^6^ Institutul de Boli Infectioase Bucharest Romania; ^7^ D‐SOLVE Consortium, an EU Horizon Europe Funded Project (No 101057917) Hannover Germany; ^8^ Spitalul Clinic de Boli Infectioase Si Timisoara Romania; ^9^ Dicle University Medical Hospitals Diyarbakir Turkey; ^10^ Ege University Medical Faculty Hospital Izmir Turkey; ^11^ Uludağ University Medical School Bursa Turkey; ^12^ Department of Medicine University Hospital, Goethe‐University Frankfurt Germany; ^13^ Heinrich Heine University Düsseldorf Germany; ^14^ Petrus Hospital Wuppertal Germany; ^15^ Department of Gastroenterology, Diabetology and Hepatology University Hospital Brandenburg, Medical School (Theodor Fontane) Brandenburg Germany; ^16^ Health Sciences, Brandenburg University of Technology Cottbus—Senftenberg, the Brandenburg Medical School Theodor Fontane and the University of Potsdam Potsdam Germany; ^17^ First Department of Gastroenterology Medical School, National and Kapodistrian University of Athens, General Hospital of Athens “Laiko” Athens Greece; ^18^ German Center for Infectious Disease Research (DZIF); Partnersite Hannover—Braunschweig Hannover Germany; ^19^ Cluster of Excellence RESIST (EXC 2155) Hannover Medical School Hannover Germany

**Keywords:** alanine aminotransferase, beneficial flares, combination therapy, delta, HDV, hepatitis flares, HIDIT‐II, interferon, randomised trial, virological outcome

## Abstract

We analysed the frequency, severity and impact of hepatitis flares in a large Phase 2 study investigating pegylated interferon‐alfa‐2a (PEG‐IFNa) for the treatment of hepatitis D. In the HIDIT‐II study, 120 patients were treated for 96 weeks with PEG‐IFNa (180 μg weekly) in combination with tenofovir disoproxil fumarate (TDF, 300 mg once daily) or placebo. Hepatitis flares were defined as ALT increases above 10 times the upper limit of normal or increases of more than 2.5‐fold above baseline or nadir values. ALT flares occurred in 28 patients (23%) during treatment (< 96) and in 14 patients post‐treatment until follow‐up Week 24. There were no differences in the flare frequency between the two treatment arms (12 PEG‐IFNa + placebo vs. 16 PEG‐IFNa + TDF). The frequency of ALT increases did not differ between cirrhotic and noncirrhotic patients. None of the patients with cirrhosis experienced liver decompensation during or after a flare. Fifty‐four per cent of the patients with ALT flare experienced a decrease in HDV RNA (> 1 log10 cop/ml) during subsequent study visits. Mean ALT levels early during treatment were higher in patients with HBsAg loss at follow‐up Week 24. More than a third of hepatitis D patients undergoing PEG‐IFNa therapy may experience ALT flares during or after treatment. ALT flares in this study posed no obvious safety risk to patients and should not lead to premature withdrawal from treatment. If ALT flares may be beneficial in single patients requires further investigation.

Clinical Trial Registration: NCT00932971, EudraCT 2008–005560‐13.

## 1 Introduction

Alanine aminotransferase (ALT) flares, defined as a sudden increase in ALT levels, are well known to occur frequently during interferon (IFN) based therapies in patients with hepatitis C and hepatitis B [1, 2, 3, 4, 5]. IFN‐induced ALT flares are described as a consequence of the immunological stimulation of interferon, which leads to increased natural killer cell activation and cytolytic T‐cell activity [3, 6, 7].

ALT flares have been described in untreated hepatitis B mono‐infected patients [8, 9], as well as during or after antiviral therapy. In PEG‐IFNa‐treated patients, ALT flares occur in 25%–40% of patients, mostly in hepatitis B e antigen (HBeAg)‐negative patients, during the 2nd–3rd month of therapy [3, 9, 10, 11].

Hepatitis D, which only occurs as either a coinfection or superinfection with hepatitis B virus (HBV) is considered the most severe and rapidly progressing form of viral hepatitis, leading to advanced liver disease and the development of cirrhosis and hepatocellular carcinoma (HCC) [12]. Although Hepatitis D is considered an orphan disease, it poses a major health burden in some regions. Approximately 5% of 240 million monoinfected individuals are coinfected with HDV [13].

Pegylated interferon alpha (PEG‐IFNa) and bulevirtide are the only treatment options for patients with chronic hepatitis D infections [14], with a variable virological response rate of 17%–47% for IFN [15, 16, 17].

In the largest randomised treatment trial investigating PEG‐IFNa in hepatitis D, the Hep‐Net‐International‐Delta‐Hepatitis‐Intervention‐study II (HIDIT‐II) 120 patients were treated with PEG‐IFNa and tenofovir (TDF) or placebo for 96 weeks. The primary endpoint, HDV RNA negativity at the end of treatment, was achieved in 48% of patients in the PEG‐IFNa plus TDF group and in 33% of the PEG‐IFNa plus placebo group [16]. Of note, ALT flares were observed during and after treatment in the patient cohort. To analyse the role and consequences of ALT flares in patients with hepatitis D and evaluate their correlation with treatment outcomes for HDV‐infected patients, we conducted a thorough analysis of the ALT flares in 120 patients enrolled in the HIDIT‐II study.

## 2 Methods

### 2.1 Study Population

We here performed a subanalysis of the patients included in the HIDIT‐II trial (EudraCT 2008–005560‐13). Initially, 120 chronic hepatitis D patients were included in the study. All patients were treated for 96 weeks with PEG‐IFNa (180 μg once weekly) and tenofovir dipivoxil (TDF) (300 mg daily) or placebo. Patients were observed for 24 weeks after the end of treatment (EOT). Details of the HIDIT‐II study with inclusion and exclusion criteria have been described previously [16].

### 2.2 ALT Flares Definition

Alanine aminotransferase flares were defined as increases above 10 times the upper limit of normal or increases of more than 2.5‐fold above baseline or nadir values. Patients were grouped into patients with ALT flares during treatment (until Week 96) (Group I), patients with ALT flares after treatment (> 96 until Week 120) (Group II) and patients without ALT flares (Group III). Patients who experienced multiple flares during and after treatment were grouped into Group I.

### 2.3 Responses and Consequences of ALT Flares

Virological responses related to ALT flares were defined as a decline or increase during the next two visits after the flares. Cut‐offs for virological responses were defined as an HDV RNA decline of more than 1 log or an HBsAg change of more than 0.5 log.

Direct clinical consequences of the ALT flares were defined as follows: Dose reduction of the study medication, bilirubin elevation > 3 mg/dL and INR elevation above 1.5 during the flares.

### 2.4 Statistics

Statistical analysis was performed using standard statistical methods (GraphPad 6.0 [GraphPad Software]). All parameters were described as mean +/− SD. *p*‐Values < 0.05 were considered statistically significant. Continuous parametric variables were analysed by *t*‐tests or ANOVA. A chi‐square test was calculated for the comparison of discrete variables. In case of an expected cell count < 5, Fisher's exact test was used instead.

### 2.5 Ethics

Details of the study design and patient recruitment are given in [16]. All patients provided written informed consent before enrolment. The HIDIT‐II study protocol conforms to the ethical guidelines of the 1975 Declaration of Helsinki as reflected in a priori approval by the ethics committee.

## 3 Results

### 3.1 Treatment Arms and Baseline Characteristics

**FIGURE 1:**
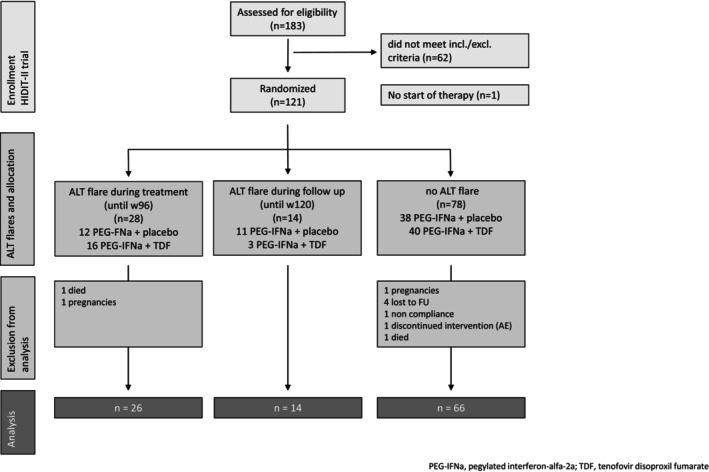
Flowchart of the study.

From all HIDIT‐II patients (*n* = 120; 59 PEG‐IFNa/TDF and 61 PEG‐IFNa/placebo), 28 patients experienced ALT flares until treatment Week 96 (Group I), 21% (*n* = 12) of the PEG‐IFNa TDF‐treated patients and 26% (*n* = 16) of the PEG‐IFNa placebo‐treated ones (*p* = 0.52; OR 0.72 95% CI 0.31–1.69). Fourteen per cent (*n* = 4) of the patients who experienced an ALT flare during treatment underwent further flares after the end of the treatment. Two patients were excluded from the analysis as they did not complete the 96‐week treatment (Figure 1). Fourteen further patients experienced an ALT flare after the end of treatment between Weeks 96 and 120. Here, ALT flares occurred more often in the PEG‐IFNa placebo arm, 18% (*n* = 11) versus 5% (*n* = 3) in the TDF arm (*p* = 0.04; OR 0.24 95% CI 0.06–0.92).

Overall, 73.1% of the patients who experienced ALT flares during treatment and 50% of those patients with ALT flares after treatment were male with a mean age of 38.3 or 41.5 years. Table 1 displays all baseline characteristics of the analysed group with no significant differences between the analysed groups and the overall HIDIT‐II population (ANOVA test). Individual characteristics of all 26 patients with ALT flares during treatment are shown in the supplement (Table S1).

**TABLE 1:** Baseline Characteristics.

	Total HIDIT‐II (*n* = 120)	Patients with ALT flares during treatment (Bl–w96) (*n* = 26)	Patients with ALT flares after treatment (> w96–w120) (*n* = 14)	Patients without ALT flares (*n* = 66)	*p*
Age—yr					
Mean (SD)	40 (11)	38 (12)	42 (11)	40 (11)	0.68
Range	20–64	20–60	20–63	21–62	
Male gender—no.(%)	79 (66)	19 (73)	7 (50)	46 (70)	0.14
HBV DNA					
Negative—no./total no. (%)	9 (7.5)	1 (3.8)	1 (7.1)	7 (11)	0.56
< 100 UI/ml—no. (%)	54 (45)	13 (50)	8 (57)	32 (49)	0.84
> 2000 UI/ml—no./total no. (%)	24 (20)	6 (23)	1 (7.1)	15 (23)	0.39
HDV RNA					
≤ 300 copies/ml no. (%)	4 (3.3)	3 (12)	0 (0)	1 (1.5)	0.06
Mean SD—log_10_ IU/ml (SD)	5.0 (1.1)	4.5 (1.2)	5.2 (1.2)	5.0 (1.3)	0.19
> 10^5^ copies/ml—no. (%)	65 (54)	17 (65)	12 (86)	40 (61)	0.20
HBsAg					
Mean (SD)—log_10_ IU/ml	3.8 (0.6)	3.9 (0.6)	3.8 (0.5)	3.8 (0.6)	0.76
< 1000 IU/mL—no. (%)	11 (9.2)	2 (7.7)	2 (14)	7 (101)	0.80
Alanine aminotransferase					
Mean (SD)—IU/l	116 (83)	96 (56)	119.6 (77)	126 (94)	0.32
> 5*ULN—no. (%)	11 (9.2)	0 (0)	2 (14)	8 (12)	0.16
Cirrhosis—no. (%)	48 (40)	11 (42)	7 (50)	23 (35)	0.51

Abbreviations: Bl, baseline visit; wk., week.

### 3.2 Natural Course and Diversity of ALT Flares

**FIGURE 2:**
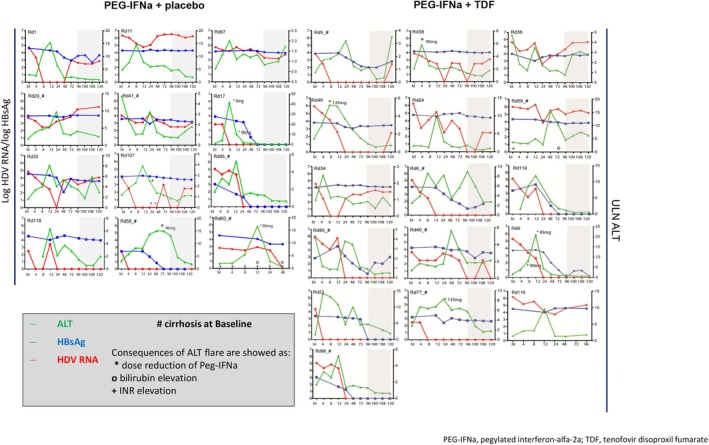
Individual courses of ALT, HDV RNA and HBsAg for the patients with ALT flares under treatment.

Three types of ALT flares (10× ULN; 2.5 > BL and 2.5 > nadir) were defined in this analysis. Figure 2 shows the individual courses of ALT, HDV RNA and HBsAg in 26 patients of Group I. Courses of the 14 patients of Group II are displayed in the Supporting Information (Figure S1). Three of the 14 patients in Group II underwent retreatments at the physician's discretion (1 with IFN/1 with TDF and 1 with PEG‐IFNa + TDF). However, ALT flares in these patients occurred before PEG‐IFNa retreatment. We observed a high diversity in the kind and time point of ALT flares within the analysed groups, reaching from one single flare at one defined time point (e.g., Patients Rd1, Rd17, etc) to sustained flares (e.g., Patients Rd58 and Rd77) or multiple flares, such as in Patient Rd6 or Rd30. Patients with multiple flares were all male; 4/7 had cirrhosis at baseline, but the numbers are too small to draw conclusions from this. Only four patients (Rd5, Rd30, Rd36 and Rd97) (all male) had both on‐ and off‐treatment flares. One of four of these individuals had liver cirrhosis, and none of these patients had HBsAg loss (see Figure 2 and Table S1).

A preliminary investigation of all patients who exhibited an ALT flare revealed a marginal tendency towards earlier onset among women compared to men. However, the number of women in the group is too small to prove this statistically (Table S1). The diversity and timing of these flares highlight the complexity and variability of ALT activity in these patients. Laboratory findings due to ALT flares, such as bilirubin elevation, were observed in 2 of the 26 patients (Figure 2). Therapy adjustments took place in eight patients, also shown in Figure 2. None of the patients developed hepatic decompensation or liver failure associated with the ALT flares.

### 3.3 Impact of ALT Flares on the Therapy Outcome

Among the 26 ALT flare patients, 16 (61%) were HDV RNA negative at EOT and still 11 (42%) at follow‐up Week 120. Four patients (15%) showed HBsAg loss at EOT.

**FIGURE 3:**
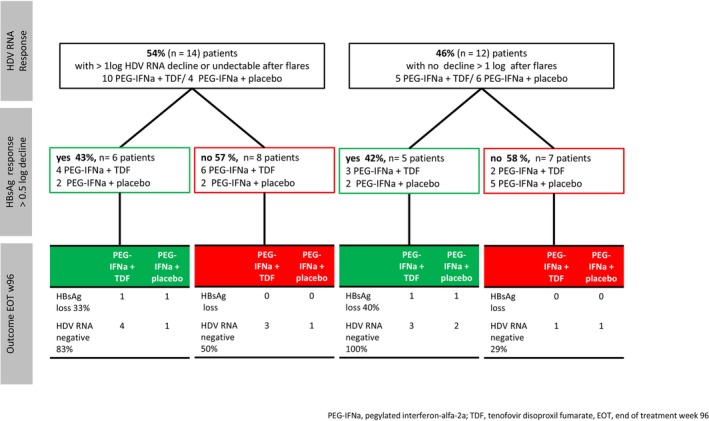
Virological outcome for patients with ALT flares during treatment (*n* = 26).

To determine if an ALT flare directly impacts viral load, we analysed the values of the three visits subsequent to the flares. Fourteen (54%) patients experienced a decline of more than 1 log HDV RNA after the flares. Of these 14 patients, only six (43%) displayed a more than 0.5 log decline in HBsAg in addition (Figure 3).

**FIGURE 4:**
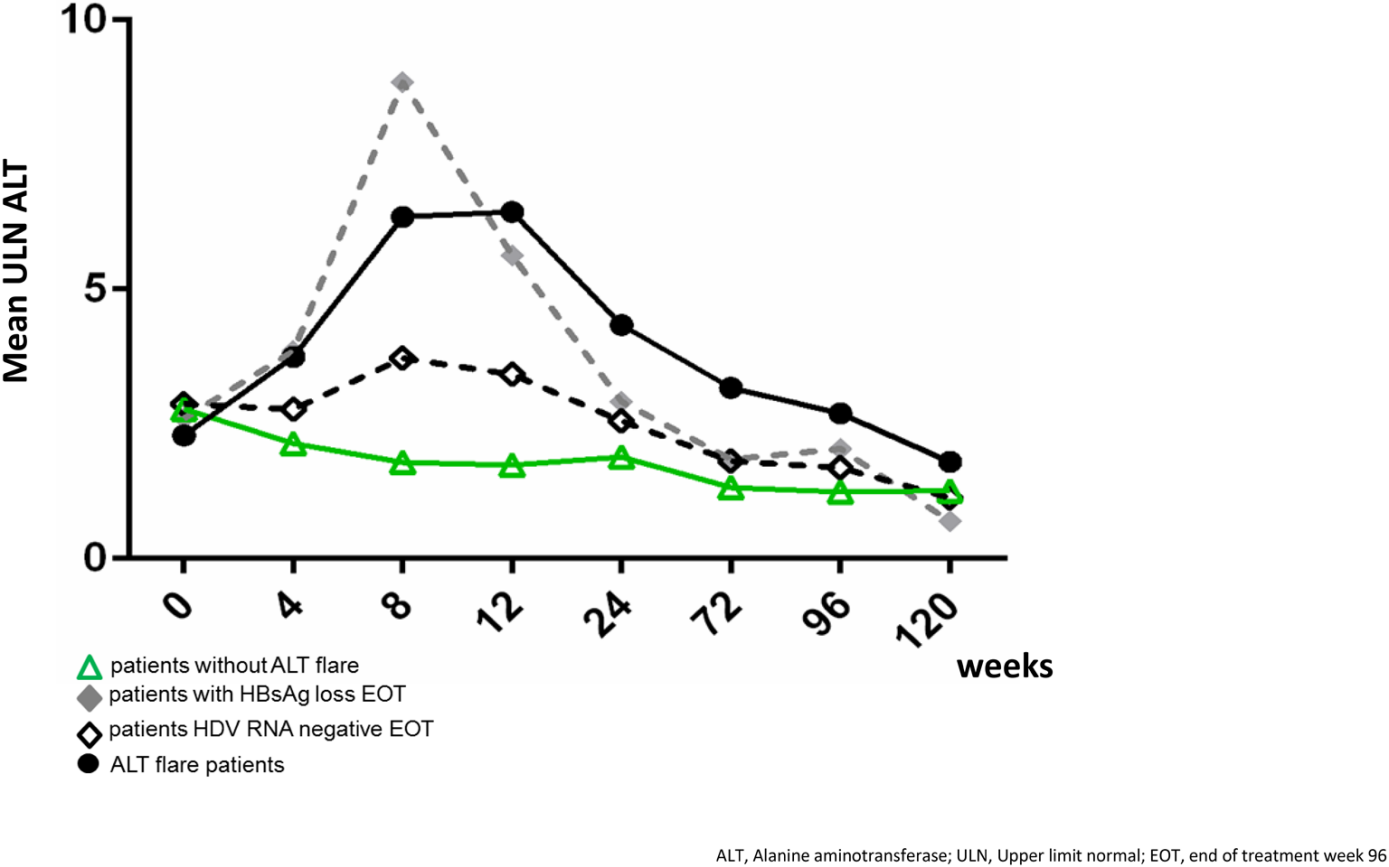
ALT course stratified for patients according to virological outcome.

Furthermore, there was no significant difference in HDV RNA (*p* = 0.15) or HBsAg (*p* = 0.29) levels at EOT or FU120 between flare and nonflare patients. Interestingly, comparing the time point and magnitude of the ALT flares of patients who cleared HDV RNA or HBsAg at EOT with pooled flare patients, we were able to show that patients with HBsAg loss at EOT showed an early (around Week 8) and higher ALT flare (Figure 4). Patients with HBsAg loss at EOT showed at Week 8 of therapy mean ALT ULN values of 16.57 (range 2.6–41.74) and patients without HBsAg loss 4.48 (range 0.89–14.60, *p* = 0.03).

### 3.4 ALT Flares in Cirrhotic Patients

In the HIDIT‐II population, liver cirrhosis at baseline was present in 48 (41%) patients [16]. In the here analysed patient cohort, flare frequency (including posttreatment flares) was nearly similar in patients with 44% (18/41) or without cirrhosis, 34% (22/65) (*p* = 0.57). Treatment adjustment or bilirubin elevation was not more frequent in flare patients with cirrhosis. Importantly, ALT flares did not lead to prolonged hepatic impairments or decompensations in any case, as described previously [16].

Notably, the cirrhotic patients experienced the ALT flares significantly later; 8/10 patients after Week 12 than the noncirrhotic patients, 4/11 (*p* = 0.009); see Figure S2.

## 4 Discussion

ALT flares are a common phenomenon during or after treatment with Type I interferons in patients with HBV infection. ALT flares have not been studied in detail in HBV/HDV coinfected patients treated with PEG‐IFNa. We have analysed the frequency, severity and impact on virological outcomes of ALT flares in the so far largest, international, multicentre clinical trial investigating PEG‐IFNa‐2a treatment in hepatitis D patients—the HIDIT‐II clinical trial [16]. The key findings of this analysis are (i) that ALT flares may occur in approximately 25% of patients in the first 24 weeks of PEG‐IFNa treatment, and in an additional 10%–15% of patients during follow‐up after treatment, (ii) that these ALT flares did not result in any liver failure—even in patients with cirrhosis, but (iii) that ALT flares may be considered as beneficial as more than half of the patients showed a HDV RNA decline within two visits after an ALT flare, and some patients who experienced flares also lost HBsAg.

About a quarter of HBV patients experience ALT flares during or after treatment with PEG‐IFNa [2, 4, 18]. This frequency appears to be comparable to what we observed in the HIDIT‐II study for patients who were coinfected with HBV and HDV. Although we did not find baseline differences between flare and nonflare patients, our results are consistent with the time point of ALT flares in HBV mono‐infected patients and their potential beneficial impact on HbsAg loss [4]. However, ALT flares were rarely observed in the first HIDIT‐I trial [15]. Given the comparable rates of flares detected in both treatment arms, this unforeseen alteration cannot be explained by the different anti‐HBV nucleotides used in HIDIT‐II (tenofovir dipivoxil) versus HIDIT 1 (adefovir). The frequency of on‐treatment and off‐treatment ALT flares in the context of treatment with PEG‐IFNa seems not to differ substantially between HDV coinfected patients and individuals with hepatitis B alone.

Importantly, in the HIDIT‐II trial, ALT flares did not lead to any hepatic decompensation or liver failure, even in cirrhotic patients. Still, dose adjustments of PEG‐IFNa according to the label must be performed and we strongly recommend that management of PEG‐IFNa therapy should be managed by experienced physicians, in particular if there is evidence of portal hypertension [14]. The presence of compensated liver cirrhosis should not be an exclusion criterion for PEG‐IFNa therapy for hepatitis D. In the HIDIT‐II trial, cirrhosis was not a negative response factor [16]. Similarly, also in HBV monoinfection, patients with liver cirrhosis respond equally well to PEG‐IFNa therapy [19].

Despite extensive discussion, the underlying causes of ALT flares during PEG‐IFNa therapy remain elusive. One theory suggests that PEG‐IFNa treatment led to the activation of NK cells. The frequency of distinct NK cell subpopulations and phenotypic and functional changes during IFN therapy was associated with the response to the treatment of hepatitis D with PEG‐IFNa [20]. In the context of PEG‐IFNa therapy for hepatitis B monoinfection, boosting innate immune responses has also been shown [21]. Boosted virus‐specific T‐cell responses leading to ALT flares have also been discussed as an alternative explanation. However, this has not yet been confirmed for PEG‐IFN treatment in either HCV or HBV patients.

The data presented here are significant in the current landscape of combination therapies involving PEG‐IFN for hepatitis D. In July 2023, the PEG‐IFN Lambda Phase 3 clinical trial was stopped after a safety review by the DSMB due to frequent ALT flares and high numbers of decompensated patients. Additionally, because of the synergistic way that IFN and Bulevirtide work together, it is vital to understand ALT flares [22, 23]. Combinations with Bulevirtide are not only discussed but also combinations with other compounds like siRNAs have been investigated in HBV‐infected patients with great success. Given the growing enthusiasm for PEG‐IFN as a companion therapy, it is of paramount importance to investigate the safety aspects of ALT flares. The mode of action between different hepatitis D treatments might be a factor in the ALT flare induction. It has been demonstrated that ALT flares were beneficial in the treatment of hepatitis D with the REP2139 compound together with PEG‐IFN [24]. Whereas, in the REEF‐D clinical trial, ALT flares that occurred during the treatment with siRNA against HbsAg were associated with HDV RNA rebound that resulted in early treatment discontinuation in eight patients [25]. The impact and safety of a hepatitis flare must be judged by the distinct mode of action.

There are obvious strengths and limitations to this analysis. The assessment of ALT flares within a large prospective, multicentre trial from different countries in Europe, the investigation of a population with a significant number of patients with cirrhosis and centralised lab testing are some of the strengths. There are limitations that should be named, such as the retrospective nature of the analysis, the limited overall number of patients in the distinct subgroups and the lack of detailed immunological or cell death markers analysis explaining flares [26]. It can be argued that a 1 log decrease in HDV RNA is too little to be considered a significant change, particularly when it comes to HDV RNA assays' widely varying sensitivities [27]. In order to identify patients who have a virological response to IFN‐based therapy, the relative log drop in viral loads is still very comparable.

In summary, PEG‐IFNa is still one of the main cornerstones of the treatment approaches for hepatitis D. ALT flares may occur during the treatment, but should not lead to premature discontinuation of PEG‐IFNa treatment. The treatment management of hepatitis D patients still needs to consider ALT flares. Further investigation is needed to determine the frequency and severity of flares as well as the relationship with late‐onset flares in cirrhotic patients, especially in the context of combination therapies with other novel antiviral approaches.

## Author Contributions

The study was designed and the protocol was written by S.H., C.Y., H.W., M.P.M. and M.C.; the study was coordinated by S.H.; patients were recruited and treated by A.C., M.G.C., K.Y., U.S.A., S.G., S.Z., A.E., S.L., G.V.P., K.P. and C.Y.; data analysis and statistics were performed by S.H. and H.W.; drafting of the manuscript was done by S.H., J.K. and H.W.; and critical revision of the manuscript was performed by all authors. S.H. has access to all data and can vouch for the integrity of data analysis.

## Ethics Statement

All procedures performed in studies involving human participants were in accordance with the ethical standards of the institutional and national research committee (Ethics Committee Hannover Medical School, Nr.5292 M) and with the 1964 Helsinki Declaration and its later amendments or comparable ethical standards.

## Consent

Informed consent was obtained from all individual participants who were included in the study.

## Conflicts of Interest

S.H. declares that she has no Conflicts of Interest. C.Y. reports personal fees from AbbVie, Gilead, Eiger and Roche. Florin A. Caruntu declares a grant/research support from HORIZON‐HLTH‐2021‐Disease‐04‐07. M.G.C. declares that she has no conflict of interest. K.Y. declares that he has no conflict of interest. U.S.A. declares that he has no conflict of interest. S.G. declares that he has no conflict of interest. S.Z. declares function as consultancy and/or speaker's bureau: Abbvie, BioMarin, Boehringer Ingelheim, Gilead, GSK, Ipsen, Madrigal, Merck/NSD, Novo Nordisk and SoBi. A.E. declares that he has no conflicts of interest. S.L. declares that he has no conflict of interest. G.V.P. declares that he has served as advisor and/or lecturer for Abbvie, Albireo, Amgen, AstraZeneca, Genesis, Gilead, GlaxoSmithKline, Ipsen, Janssen, Novo Nordisk, Roche and Takeda and has received research grants from Abbvie, Gilead and Takeda. K.P. declares that she has no conflicts of interest. M.P.M. reports a research grant from Merck, personal fees from Abbvie, Assembly Bio, BMS, Gilead, GSK, Merck and Precision Biosciences, and is the principal investigator in clinical trials for Merck. M.C. reports personal fees from AbbVie, AiCuris, Gilead, GlaxoSmithKline (GSK), Janssen, Merck/MSD, Novartis, Roche and Swedish Orphan Biovitrum (SOBI) Falk. J.K. declares that she has no conflicts of interest. H.W. reports research grants from Abbvie, Biotest, Gilead and HORIZON, personal fees from AbbVie, Aligos, Altimmune, Biotest, BMS, BTG, Dicerna, Enanta, Gilead, Janssen, Merck/MSD, MYR GmbH, Roche, Vir Biotechnology, Intercept, Falk, Norgine and Pfizer, serves as principal investigator in clinical trials for Abbvie, Altimmune, Bristol‐Myers Squibb, Gilead, Janssen, MYR GmbH, Novartis and Vir Biotechnology and holds a role in the DGVS executive board.

### Material From Other Sources

No material from other sources was used.

## Supporting information


Data S1.

## Data Availability

The data that support the findings of this study are available from the corresponding author upon reasonable request.
