# Supplementary material for: Frequency, Severity and Impact of Pegylated Interferon Alpha–Associated Flares in Hepatitis D Infection
Source: J Viral Hepat. 2025 Mar 15;32(4):e70022. doi: 10.1111/jvh.70022 (PMC11909584; doi:10.1111/jvh.70022)
Supplement: Supplementary file 1 — Data S1. [file JVH-32-0-s001.docx]

**Frequency, severity and impact of pegylated interferon alpha associated flares in hepatitis D infection**

Svenja Hardtke, Cihan Yurdaydin Florin A Caruntu, Manuela G. Curescu, Kendal Yalcin, Ulus S. Akarca,Selim Gürel, Stefan Zeuzem, Andreas Erhardt, Stefan Lüth, George V. Papatheodoridis, Kerstin Port, Michael P. Manns , Markus Cornberg, Julia Kahlhöfer and Heiner Wedemeyer for the HIDIT-2 study team

**Table of Content**

Supplemental Table 1: Individual characteristics of patients with ALT flares during treatment (n=26) 2

Supplemental Figure 1: Individual courses of ALT, HDV RNA and HBsAg for the patients with ALT flares after End of treatment week 96 3

Supplemental Figure 2: Time of occurrence of ALT flares in cirrhotic and non-cirrhotic patients at baseline. 4

# **Supplemental Table 1: Individual characteristics of patients with ALT flares during treatment (n=26)**

| **ID** | **sex** | **Arm#** | **HDV RNA IU/ml at baseline** | **HDV RNA IU/ml**  **at w96** | **HDV RNA IU/ml at week 120** | **ALT ULN**  **at baseline** | **ALT flare category:**  1 ALT > 10 ULN  2 ALT > 2.5 above Nadir  3 ALT > 2.5 above Bl | **Timepoint of Flare week** multiple^$^ on and off treatment° | **Cirrhosis baseline** | **HDV RNA drop > 1 log after flare** | **HbsAg decline or increase >0.5 log**  **after flares** | **HBsAg loss EOT** | **HBV genotype** |
| --- | --- | --- | --- | --- | --- | --- | --- | --- | --- | --- | --- | --- | --- |
| 1 | female | placebo | 49600 | 426 | 747 | 2,94 | 1 and 3 | 8-12 |  |  |  |  | nd |
| 5 | male | verum | 6864 | 0 | 300 | 0,78 | 3 | 24, 120^$,°^ | Yes |  | decline |  | E |
| 6 | male | verum | 116000 | 0 | 0 | 2,56 | 3 | 8,24^$^ | Yes | Drop LoQ^+^ | decline |  | D |
| 9 | female | verum | 199000 | 0 | 0 | 1,71 | 1 and 3 | 8-12 |  | Yes | decline |  | nd |
| 11 | male | placebo | 2098000 | 3316000 | 1641000 | 4,91 | 1 and 3 | 8 |  |  |  |  | D |
| 17 | female | placebo | 665 | 0 | 0 | 1,26 | 1 and 3 | 8-12 |  |  | decline | yes | D |
| 23 | male | verum | 25110 | 0 | 0 | 1,67 | 1 and 3 | 4-12,48^$^ |  |  | decline | yes | nd |
| 24 | male | verum | 1809000 | 0 | 300 | 1,68 | 1 and 3 | 24 |  | Yes |  |  | D |
| 29 | female | placebo | 5540 | 75880 | 163300 | 1,97 | 3 | 12-24 | yes | Increase* |  |  | nd |
| 30 | male | placebo | 70850 | 1276 | 11540 | 1,69 | 3 | 12,120^$,°^ |  | Drop LoQ^+^ | decline |  | D |
| 34 | male | verum | 37220 | 2703 | 2849 | 0,98 | 3 | 4 |  | Drop LoQ^+^ |  |  | D |
| 36 | male | verum | 415300 | 42870 | 437300 | 4,72 | 2 | 12,120^$,°^ |  | yes | increase |  | nd |
| 38 | female | verum | 7550 | 0 | 1206 | 2,09 | 3 | 4 |  | yes |  |  | D |
| 40 | male | verum | 4200 | 0 | 0 | 0,98 | 3 | 72 | yes | yes |  |  | nd |
| 41 | male | placebo | 10160 | 300 | 1090 | 4,76 | 2 | 24 | yes | yes | increase |  | D |
| 46 | male | verum | 865000 | 0 | 300 | 1,51 | 3 | 8-12 |  | yes | decline |  | nd |
| 58 | male | placebo | 300 | 0 | 0 | 3,38 | 1 and 3 | 8-24,24-96^$^ | yes |  | decline |  | D |
| 59 | male | verum | 993300 | 102500 | 154200 | 3,83 | 1 and 3 | 72 | yes |  |  |  | D |
| 77 | male | verum | 300 | 0 | 0 | 2,93 | 3 | 24 | yes |  |  |  | D |
| 85 | female | verum | 873900 | 0 | 0 | 4,17 | 2 | 24-48 | yes |  | decline |  | nd |
| 95 | male | placebo | 117800 | 0 | 0 | 1,73 | 3 | 12 | yes | yes | decline | yes | D |
| 96 | male | verum | 14820 | 740 | 300 | 1,58 | 3 | 8-24 |  | Drop LoQ^+^ |  |  | D |
| 97 | male | placebo | 55420 | 1915 | 5511 | 0,66 | 3 | 8,120^$,°^ | yes |  |  |  | nd |
| 107 | male | placebo | 8671 | 0 | 300 | 1,20 | 3 | 12 |  |  |  |  | nd |
| 118 | male | placebo | 300 | 0 | 0 | 2,40 | 3 | 12 |  | yes | increase |  | D |
| 119 | female | verum | 127700 | 0 | 0 | 1,20 | 1 and 3 | 12 |  | yes | decline | yes | nd |

# verum = pegylated interferon-alfa-2a plus tenofovir disoproxil fumarate; placebo = pegylated interferon-alfa-2a plus placebo, * Increase after flare, + HDV RNA droped from the LoQ towards HDV RNA negativity; nd=not done


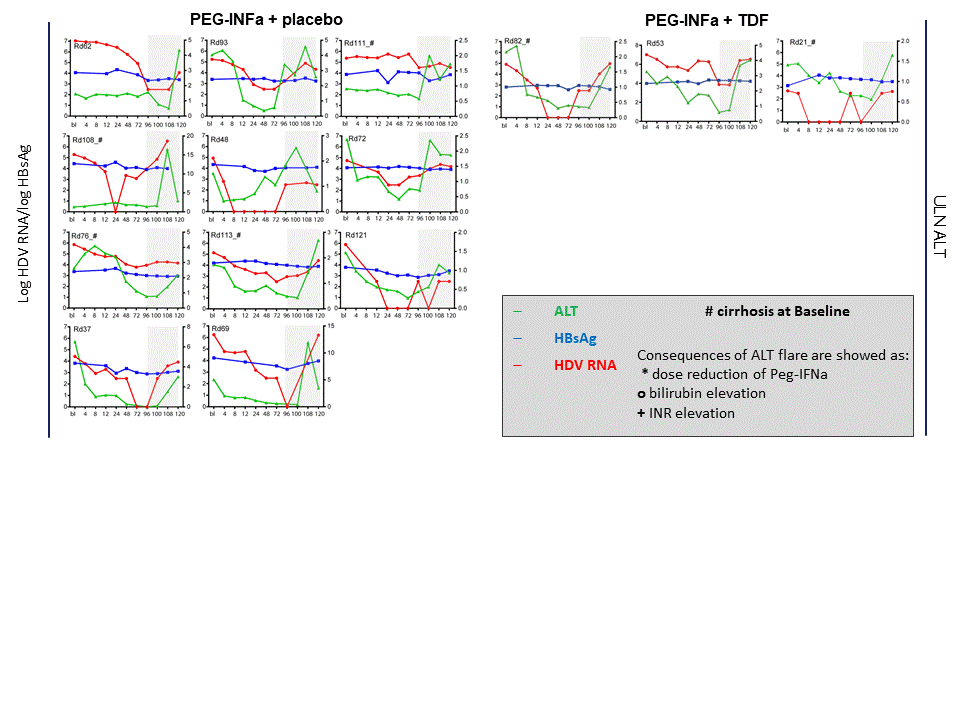


# **Supplemental Figure 1: Individual courses of ALT, HDV RNA and HBsAg for the patients with ALT flares after End of treatment week 96**

PEG-IFNa, pegylated interferon-alfa-2a; TDF, tenofovir disoproxil fumarat


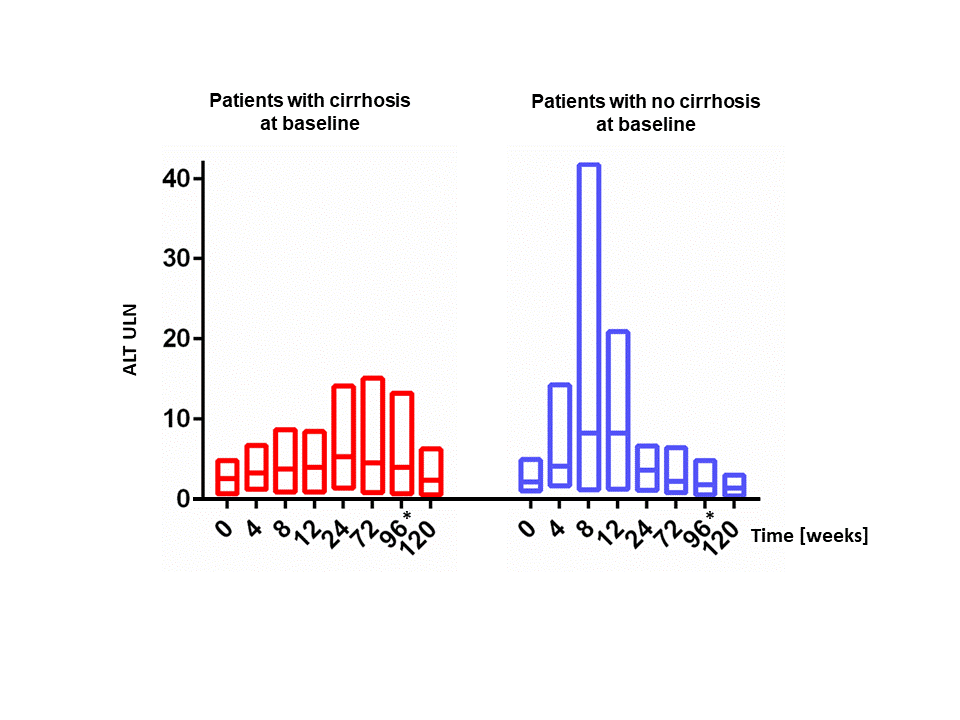


# **Supplemental Figure 2: Time of occurrence of ALT flares in cirrhotic and non-cirrhotic patients at baseline**

*End of treatment
